# 

**Published:** 1919-08-16

**Authors:** 


### Buildings Contemplated and in Progress.

Crewe.—It is proposed to extend the Cottage Hospital.

Hanwell.—As a war memorial it is proposed to enlarge the Cottage Hospital at a cost of £3,000.

Rockhampton, Queensland.—Extensive additions are to be made to Rockhampton General Hospital.

Scunthorpe.—£16,000 has been promised towards the cost of a proposed new general hospital.

Sheffield.—The venereal clinic is to be provided with better accommodation at a cost of £809.

Swadlincote.—The local War Memorial Committee has received an offer from Mr. B. Goodhead of £1,000 towards the cost of a cottage hospital for the district.

Buxton.—An extension of the Cottage Hospital, providing twenty-eight beds, an *x*-ray room, operating-theatre, etc., at an estimated cost of £4,000, as a war memorial, is proposed.

Woking.—The local war memorial will take the form of a new hospital and memorial cross. The total cost is estimated at £37,000.

### Gifts and Bequests.

MR. DAVID WHITTINGHAM, for thirty-nine years coroner for the Nottingham Division, left £50 to the Notts General Hospital.

MR. H. W. THOMPSON, Newcomb, Winscombe, Somerset, left £1,000 to King Edward's Hospital Fund and £1,000 to Winifred House Children's Convalescent Home, Tollington Park.

MR. JOSEPH WAGNER, of Hampstead, has left the London Hospital a legacy of £250.

MR. JOHN MCINTYRE, of Tynemouth, left £200 to the Fleming Memorial Hospital for Sick Children.

THE "Silver Thimble Fund" has given the Wimbledon Hospital and the Nelson Hospital £1,000 each for the endowment of a bed.

